# Decoding Femoral Vein Leiomyosarcoma: A Rare Vascular Malignancy Revealed Through Imaging

**DOI:** 10.7759/cureus.64370

**Published:** 2024-07-11

**Authors:** Shivani S Bothara, Ravishankar Patil, Pratapsingh Parihar, Rishabh Dhabalia, Abhinav Kadam, Suhit Naseri

**Affiliations:** 1 Department of Radiodiagnosis, Jawaharlal Nehru Medical College, Datta Meghe Institute of Higher Education and Research, Wardha, IND; 2 Department of Medicine, Jawaharlal Nehru Medical College, Datta Meghe Institute of Higher Education and Research, Wardha, IND; 3 Department of Pathology, Jawaharlal Nehru Medical College, Datta Meghe Institute of Higher Education and Research, Wardha, IND

**Keywords:** mri thigh, vascular soft tissue tumour, swelling in thigh, leiomyosarcoma, femoral vein

## Abstract

Lumps are commonly found in the femoral triangle. Femoral hernias and lymphadenopathy are included in the differential diagnosis. One of the rare possibilities of femoral triangle swellings is leiomyosarcoma. Leiomyosarcoma originating from the walls of blood vessels is very rare, and only a few cases are reported. We present a case of a 50-year-old male patient complaining of swelling over the left thigh. Ultrasonography showed a highly vascular soft tissue tumour in the anteromedial compartment of the thigh. Magnetic resonance imaging (MRI) was done later. It showed a well-defined, heterogeneously enhancing solid cystic lesion along the femoral vein with intravenous extension, and the femoral artery was seen encasing along its length. A surgical exploration of the lesion suggested a mass originating from the femoral vein, obstructing the vein itself. The mass was excised, and the defect in the vein was repaired. Histopathological examination revealed the mass to be leiomyosarcoma of vascular origin.

## Introduction

Femoral triangle mass is an umbrella term comprising masses from various tissues. Amongst them, rare are femoral artery aneurysms, peripheral nerve sheath tumours, hemangiopericytoma, and leiomyosarcoma. Rarely do leiomyosarcomas develop straight from blood vessels. As a result, few published case studies describe this type of malignancy's clinical manifestation and therapeutic outcomes. The tumours originate directly from a large vein or arterial muscular wall. The femoral vascular bundle is affected by most tumours that arise in the extremities [1]. Based on their place of origin, leiomyosarcomas may be broadly classified into three kinds [1]. The most prevalent type of leiomyosarcomas is those of the soft tissues [2], followed by cutaneous leiomyosarcomas, which have the best prognosis [2], and vascular leiomyosarcomas.

Any leiomyosarcoma arising from a major vessel is diagnosed based on clinical, radiological, and pathological findings [2]. Few leiomyosarcomas of vascular origin have been reported; most are solitary case reports [3-5]. The prognosis of patients with this type of leiomyosarcoma is controversial due to several factors. Some studies suggest that vascular leiomyosarcomas have a poorer prognosis than those from other locations. This is partly because of their potential for early hematogenous spread and the complexity of achieving complete surgical resection [1,6,7]. However, other reports indicate variable outcomes, with some patients responding well to treatment involving a combination of surgery and adjuvant therapies [1,6,7]. Currently, the majority of patients receive radiation therapy and surgery to preserve their limbs, much like other soft tissue sarcomas. This case highlights an unusual presentation of a 50-year-old male with a mass in the thigh region, later revealed as a leiomyosarcoma originating from the femoral vein. Very few such cases have been reported in the literature so far.

## Case presentation

A 50-year-old male patient presented to the Surgery Outpatient Department with a two-month history of swelling in his left thigh. The swelling had an insidious onset, gradually increased in size, and was not associated with pain or discharge. He had no significant medical or family history and denied any history of trauma. The patient had no comorbidities. He was admitted to the male surgery ward for further management.

On examination, the patient's pulse was 78 beats per minute (bpm), blood pressure was 130/80 mmHg, and oxygen saturation was 99% on room air. Peripheral pulses were palpated and were normal. There were no signs of pallor, icterus, clubbing, cyanosis, or pedal oedema. A local examination of the left thigh revealed that the swelling was approximately 5.6 x 4.3 cm. Upon systemic examination, the cardiovascular, respiratory, abdomen, and central nervous systems were within normal limits. All biochemical and pathological investigations were sent, described in Table 1.

**Table 1:** Laboratory investigations of the patient on admission g/dL, gram per decilitre; micron, micrometre; pg, picogram; cumm, cubic millimetre; fL, femtolitre; mg/dL, milligram/decilitre; mEq/L, milliequivalent/litre; IU/L, international units/litre; U/L, units/litre; MCHC, mean corpuscular haemoglobin concentration; MCV, mean corpuscular volume; MCH, mean corpuscular haemoglobin; RBC, red blood cell; WBC, white blood cell; RDW, red cell distribution width; APTT, activated partial thromboplastin time; INR, international normalized ratio; SGOT, serum glutamic oxaloacetic transaminase; SGPT, serum glutamic pyruvic transaminase; HIV, human immunodeficiency virus

Laboratory parameter	Results	Normal values
Haemoglobin	12.6 g/dL	11-14 g/dL
MCHC	35.4 g/dL	32-36 g/dL
MCV	89 micron	79-92 micron
MCH	31.5 pg	27-31 pg
Total RBC count	3.41 x 10^6 ^cells/cumm	2.50-5.50 x 10^6 ^cells/cumm
Total WBC count	6300 cells/cumm	4000-11000 cells/cumm
Total platelet count	2.48 x 10^6 ^cells/cumm	1.50-4.50 x 10^6 ^cells/cumm
Haematocrit	43.4%	40-54%
Monocyte	3%	2-8%
Granulocyte	56%	40-60%
RDW	14.1 fL	12.2-16.1 fL
Eosinophils	2%	1-4%
Basophil	0%	<1%
APTT	34.2 seconds	29.5 seconds
Prothrombin time	14.2 seconds	11.3 seconds
INR	1.10	1.00
Urea	18 mg/dL	6.24 mg/dL
Creatinine	0.6 mg/dL	0.59-1.04 mg/dL
Sodium	134 mEq/L	135-145 mEq/L
Potassium	4.3 mEq/L	3.5-5.1 mEq/L
Alkaline phosphate	74 IU/L	75-124 IU/L
SGOT	47 IU/L	8-45 IU/L
SGPT	38 IU/L	7-56 IU/L
Total protein	7.4 g/dL	6.0-8.3 g/dL
Albumin	4.1 g/dL	3.4-5.4 g/dL
Total bilirubin	0.6 mg/dL	0.1-1.0 mg/dL
Conjugated bilirubin	0.1 mg/dL	0.1-0.4 mg/dL
Unconjugated bilirubin	0.5 mg/dL	0.2-0.6 mg/dL
HIV card test	Negative	-

**Figure 1:**
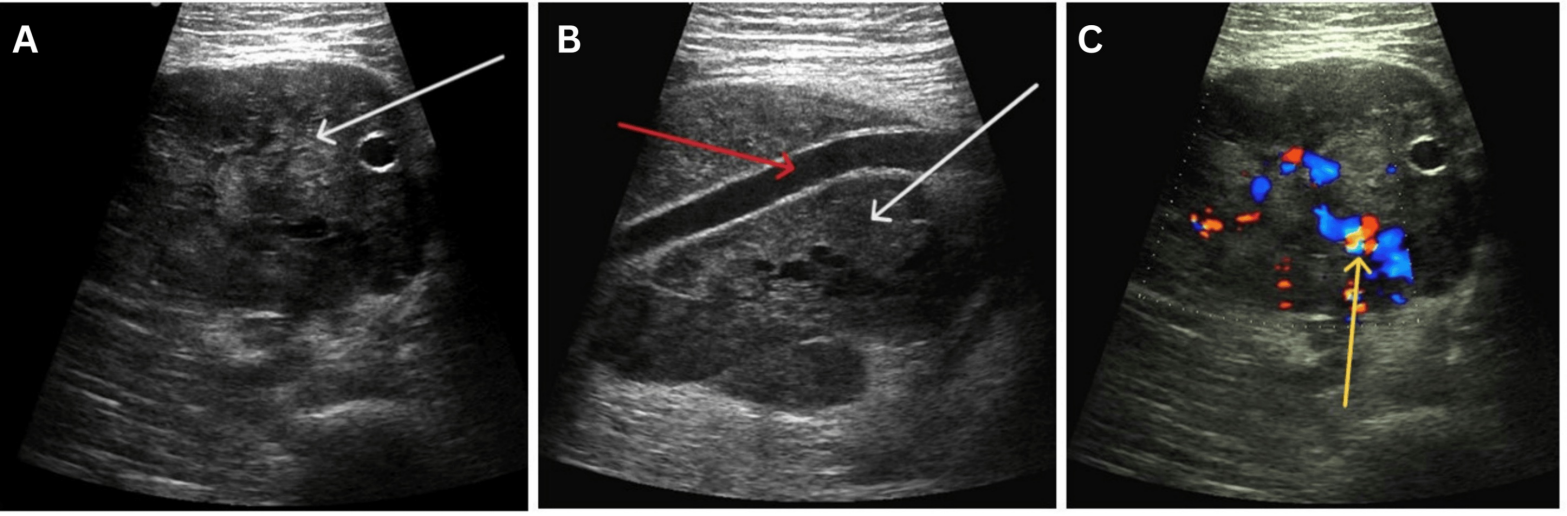
Ultrasonography (USG) of the swelling at the anteromedial compartment of the thigh revealing a soft tissue lesion (white arrow) encasing the superficial femoral artery (red arrow). A) Transverse view; B) Longitudinal view; C) Doppler USG of the same lesion shows increased vascularity (yellow arrow).

Suspecting it was a lymph node, an ultrasound at the site of swelling was done, which showed a well-defined, large, heterogeneously hypoechoic lesion with increased vascularity on Doppler, most likely a vascular soft tissue tumour in the anteromedial compartment of the thigh. The lesion was seen inside the lumen of the femoral vein for a portion and encasing the femoral artery (Figure 1).

**Figure 2:**
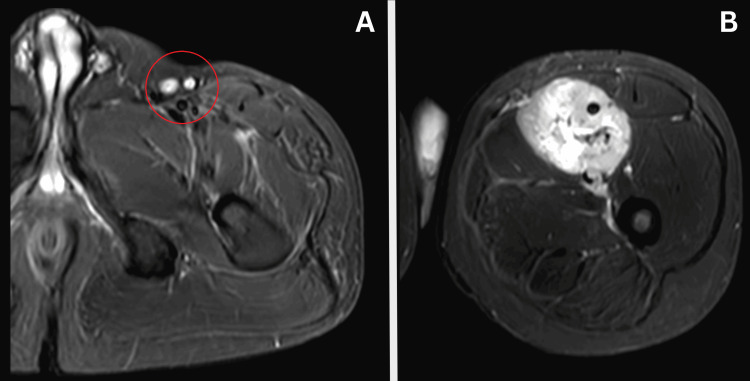
Magnetic resonance imaging (MRI) of the left thigh showing short tau inversion recovery (STIR) axial images of the swelling at different levels. A) Femoral artery, femoral vein, and femoral nerve (indicated by a red circle); B) At the inferior level, the mass is originating from the femoral vein, so the vein is not visible as it was in image A, and the lesion is encasing the femoral artery.

**Figure 3:**
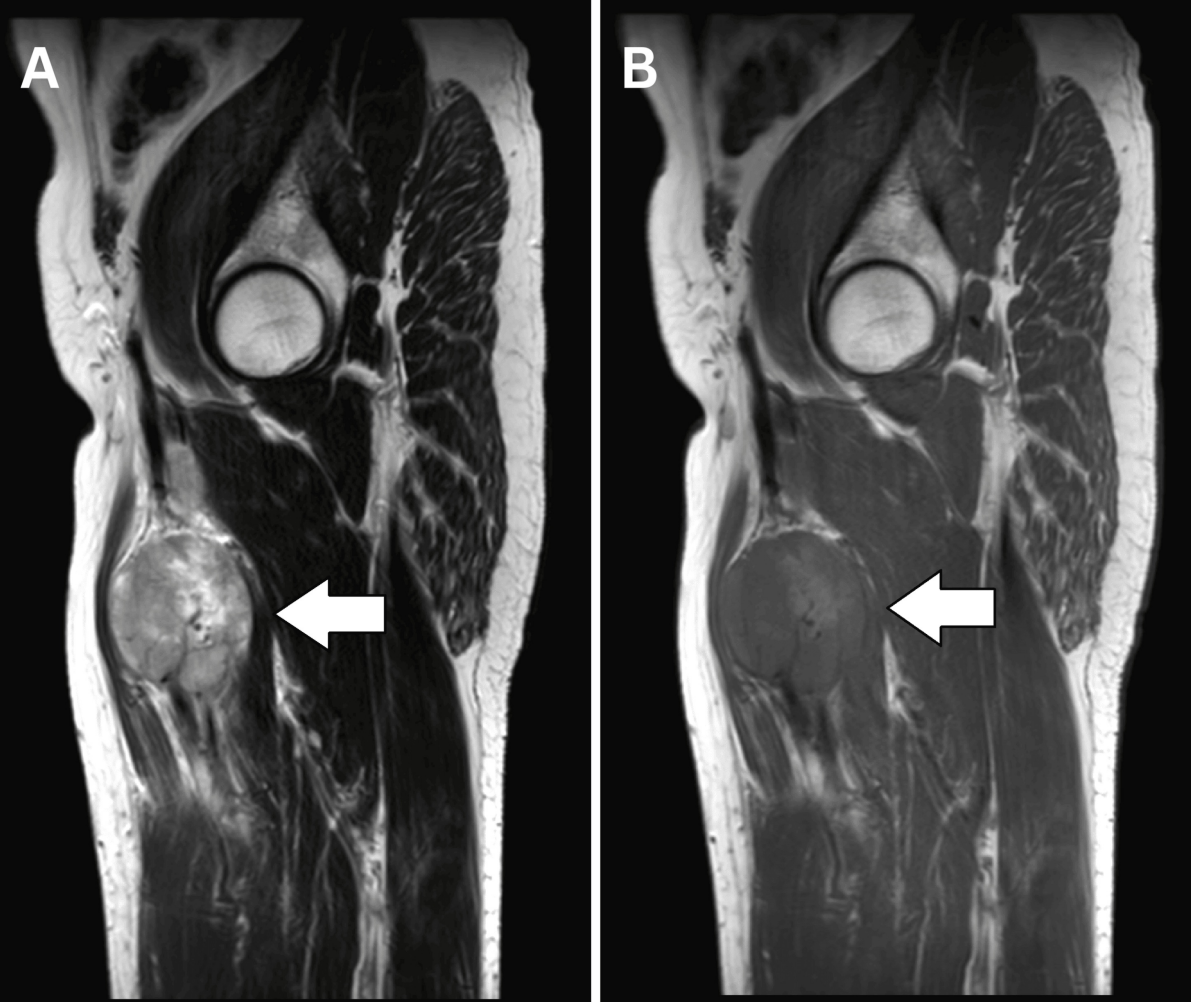
Magnetic resonance imaging (MRI) of femoral vein leiomyosarcoma. A) Sagittal T2 weighted image shows a well-defined mass lesion appearing hyperintense (white arrow); B) Sagittal T1 weighted images show a well-defined mass lesion appearing iso-intense imaging (white arrow). Lesion lies between adductor magnus medially vastus intermedius and rectus femoral laterally.

**Figure 4:**
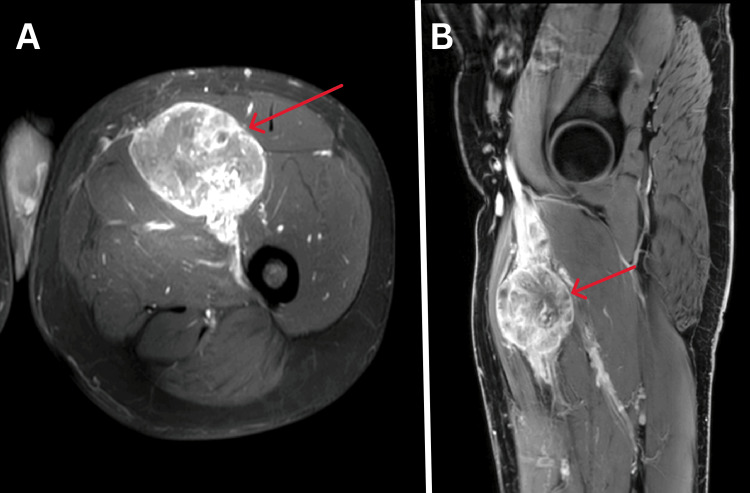
Contrast-enhanced magnetic resonance imaging (MRI) of femoral vein leiomyosarcoma. A) Axial and B) Sagittal T1 weighted MRI images show well-defined heterogeneously enhancing solid cystic lesions along with femoral vein (red arrow) and intravenous extension.

A magnetic resonance imaging (MRI) was advised for confirmation, revealing a well-defined, heterogeneously enhancing, lobulated solid cystic lesion along the femoral vein, with an intravenous extension between the adductor magnus medially and the vastus intermedius and rectus femoris laterally. The lesion encased the femoral artery along its length and appeared to arise from the smooth muscle wall of the femoral vein, according to the imaging characteristics suggestive of leiomyosarcoma (Figures 2-4).

**Figure 5:**
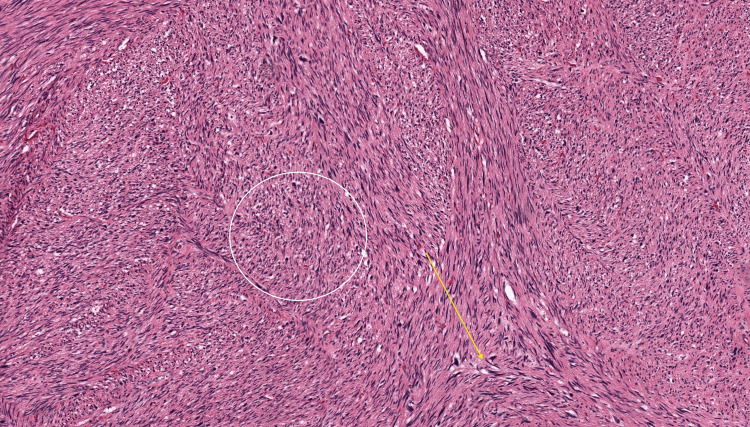
H&E, 20x section from the tumour mass shows spindle cells with intersecting fascicles with infiltrative borders (yellow arrow). These spindle cells have “cigar-shaped” nuclei, atypia, mitoses and necrosis (white circle). Histopathological features are suggestive of leiomyosarcoma. H&E, haematoxylin and eosin

Then, surgical exploration was done where the mass was seen in continuity with the femoral vein and extending into the femoral vein. Surgical excision of the mass was done, and Prolene sutures were used to repair the defect. The procedure went uneventfully. The mass was sent for histopathological examination, and it was confirmed to be leiomyosarcoma (Figure 5).

An oncologist's opinion was taken, which suggested chemotherapy for the patient. The patient was not willing to do it, and due consent was taken. The patient's condition improved eventually, and he was discharged on postoperative day 5, with a total hospital stay of seven days.

## Discussion

Leiomyosarcomas represent 5% to 6% of all sarcomas and are malignant tumours from smooth muscle [8]. Perl and Virchow initially documented them in 1871 [9]. The uterus and gastrointestinal tract are the frequent sites for leiomyosarcoma [8]. Two per cent of leiomyosarcomas are venous in origin, most frequently from the vena cava, and they arise in the smooth muscle of the vessel walls [10,11]. As far as we know, less than 30 instances of leiomyosarcomas from the femoral vein have been documented [12]. They frequently block venous flow and exhibit intraluminal tumour extension. There may also be symptoms if the nearby neuromuscular bundle is compressed.

The diagnosis uses radiological and pathological findings, with immunohistochemistry as a supplement to histology, which is the gold standard. When a clinical case or imaging points to a potential sarcoma, it is advisable to consult the oncology unit. The preferred course of therapy is wide surgical excision [13]. To reduce the size of the tumour, neoadjuvant chemotherapy may be administered. Adjuvant radiation treatment following surgery is advised [13]. Early blood-borne metastases may be the reason for the comparatively poor prognosis of leiomyosarcomas of vascular origin [8]. Every case listed by Berlin et al. had metastases, and five (83.3%) of the individuals lost their lives to metastatic illness in less than five years [1].

In our case, various differentials were initially considered, such as lymphadenopathy, fetal hernia, femoral artery aneurysm, and peripheral nerve sheath tumour. Lymphadenopathy could have been the first clinical diagnosis, but radiological investigations suggested it as a vascular soft tissue tumour, excluding lymphadenopathy. Ultrasonography also ruled out the femoral hernia, as it showed no defect in the femoral sheath. In our case, the femoral artery was seen encased by a tumour, and its lumen was normal, thus excluding the femoral artery aneurysm. A peripheral nerve sheath tumour was ruled out on an MRI, as it was seen to originate from the femoral vein and not the nerve sheath.

## Conclusions

Femoral vein leiomyosarcoma is the rarest of all sarcomas, and any swelling of the femoral triangle mass can be considered as a differential diagnosis. Generally, femoral triangle swelling is benign, but in our case, it can present as a malignant lesion and thus must be appropriately imaged through ultrasound and MRI imaging. Also, the hypoechoic region on ultrasound should be thoroughly assessed in the case of vascular sarcomas, as in our case, the lesion surrounded an artery, and the vein was involved. MRI is necessary, as it provides knowledge about location and luminal involvement. The treatment comprises surgical management and adjuvant chemotherapy in such cases.
